# Integrating Cupping Therapy in the Management of Sudden Sensorineural Hearing Loss: A Case Report

**DOI:** 10.7759/cureus.7063

**Published:** 2020-02-20

**Authors:** Zainab A Almusleh, Walid El Ansari

**Affiliations:** 1 Otolaryngology, Hamad Medical Corporation, Hamad General Hospital, Doha, QAT; 2 Surgery, Hamad Medical Corporation, Hamad General Hospital, Doha, QAT; 3 Surgery, University of Skovde, School of Health and Education, Skovde, SWE

**Keywords:** cupping therapy, case report, sudden sensorineural hearing loss (ssnhl)

## Abstract

Sudden sensorineural hearing loss (SSNHL) is most often defined as a rapid hearing loss of ≥ 30 decibels across at least three contiguous audiometric frequencies over a time of ≤ 72 hours. Cupping therapy has been practiced across the world for thousands of years. Cupping therapy is practiced by creating suction inside cups that are placed on predefined skin areas. Our case is a 48-year-old female with a four-year history of Meniere’s disease, recurrent tinnitus, episodes of dizziness attacks, and fullness of the right ear. The patient developed sudden sensorineural hearing loss. She received conventional treatment and wet cupping therapy as a complementary integrative treatment. After the integrative management protocol was completed, pure tone audiometry tests revealed significant hearing improvement across almost all frequencies. To the best of our knowledge, this case presentation is the first reported case of this type. A positive effect of cupping was reported in our case as an integrative complementary treatment. Large, well-designed quality clinical trials to evaluate the efficacy and safety of wet cupping therapy (WCT) as a complementary treatment of SSNHL is highly recommended.

## Introduction

Sudden sensorineural hearing loss (SSNHL) is a hearing loss of ≥ 30 decibels across at least three contiguous audiometric frequencies over a time of ≤ 72 hours [1]. SSNHL can be due to inner ear autoimmune disease, viral, vascular, or as a part of Meniere’s syndrome [1-2]. Ciuman reported that most of the sudden hearing loss was due to circulatory disturbances [3].

Meniere’s syndrome is a long-term progressive disease that damages the inner ear balance and hearing parts, which may lead to permanent hearing loss [4]. Whilst conventional treatment includes drugs, exercise, and diet changes, recent studies have explored some complementary and alternative medicine (CAM) therapies to alleviate the symptoms, such as acupuncture, acupressure, chiropractic, and others [5].

To date, no studies have examined the effect of cupping therapy on SSNHL or Meniere’s syndrome, despite some studies reporting the effectiveness of using wet cupping therapy (WCT) in the management of various diseases, such as migraine, shoulder pain, and neck pain [6-7]. It also may improve the quality of life of patients with chronic medical conditions [8].

Cupping therapy (Al-Hijama in Arabic) means to reduce in size [9]. It has been practiced across the world for thousands of years. It was a cornerstone of many traditional medical systems, such as traditional Chinese medicine (TCM), Unani medicine, traditional Korean medicine (TKM), and Islamic medicine. Cupping therapy is practiced by creating suction inside cups that were placed on predefined skin areas [10]. The mechanism of the action of cupping therapy has not been clear until now, but there are some theories, such as immunomodulation theory, genetic theory, and other theories, to try to explain it [10-11].

To the best of our knowledge, this case presentation is the first reported case of this type.

## Case presentation

A 48-year-old female presented with right sudden SSNHL, dizziness, intractable tinnitus, and right ear fullness at our audiology clinic. She had a four-year history of Meniere's disease, recurrent dizziness attacks, an intermittent mild sensation of imbalance usually lasting < 30 minutes, recurrent tinnitus, and fullness of the right ear.

**Figure 1:**
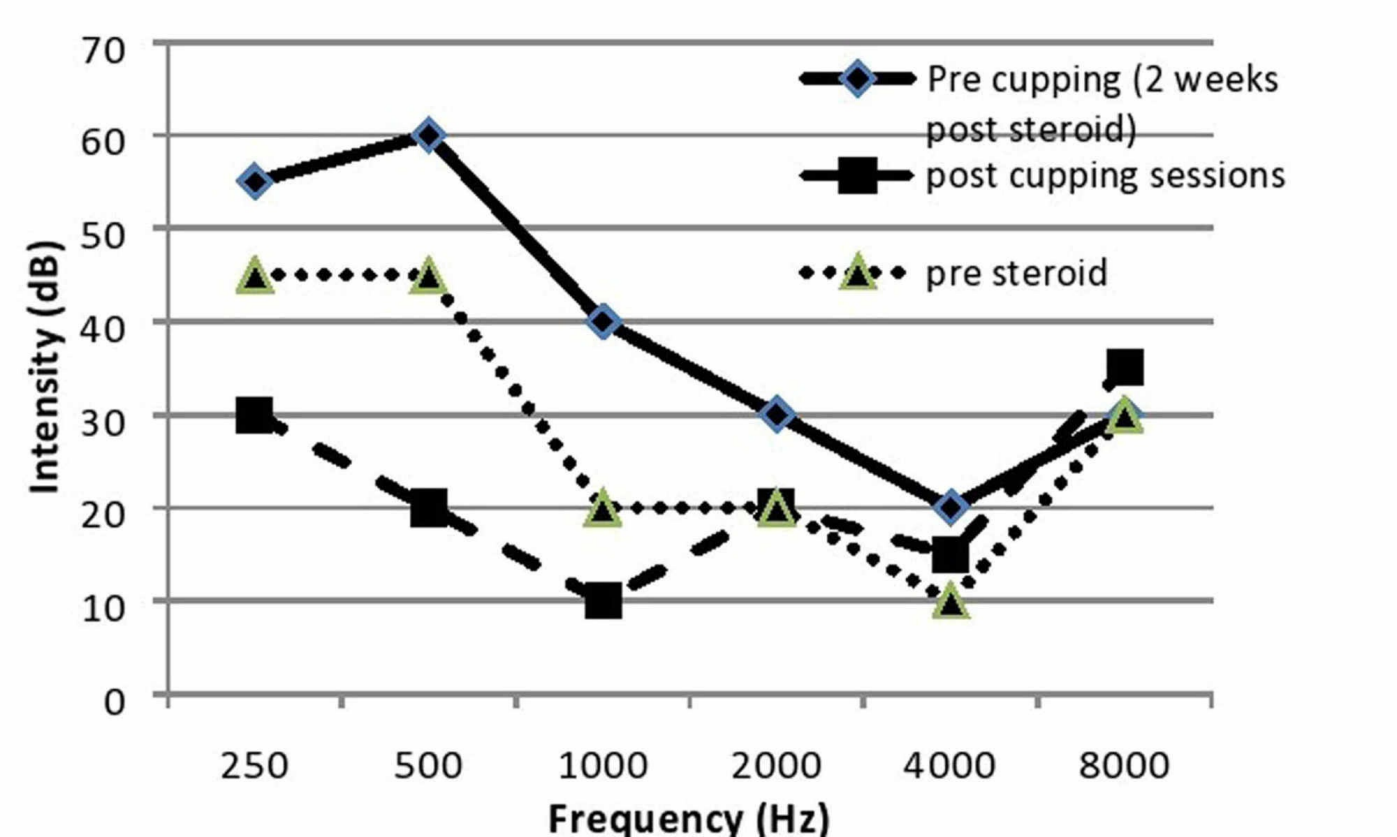
Pure tone audiometry tests

Her hearing test showed typical low-frequency SSNHL, reaching up to 45 dB (Figure 1). To identify the handicapping level of the patient due to tinnitus and dizziness, the patient answered the Dizziness Handicap Inventory (DHI) [12] and the Tinnitus Handicap Inventory (THI) questionnaires [13]. Her scores were 52 (moderate handicap), and 86 (catastrophic handicap), respectively.

A full course of prednisolone (1 mg/kg) was prescribed to the patient for two weeks, followed by a tapering dose for another two weeks, together with a diuretics course for seven days and betahistine (16 mg three times per day). The patient then presented to our clinic two weeks later with further deterioration of her hearing by 10-15 dB at low frequencies, reaching up to 60 dB.

At this stage, a decision was made to include WCT as a complementary therapy to the treatment protocol after the acceptance of the patient. Written informed consent was obtained from the patient to publish this case report.

Cupping therapy was performed by a professional cupping therapist on post-auricular cupping points. A light to medium suction pressure (between 200 and < 350 millibars) was applied for three to five minutes using an electrical suction pump [14]. Other steps taken were performing superficial scarifications, cups were reapplied, cups were removed, skin disinfection was performed, and sterile wound dressings were applied. The same procedure was followed for all six scheduled sessions (a session every two weeks). Upon completion of the treatment regime, we assessed the patient’s hearing and readministered the THI and DHI.

Pure tone audiometry tests revealed a significant hearing improvement by 25-35 dB at low frequencies (reaching to 20 dB) after completion of the WCT treatment regime. Figure 1 shows the notable deterioration of hearing following the use of steroids, followed by a significant improvement after the inclusion of WCT (which reached a normal level at frequencies: 500 Hz, 1000 Hz, 2000 Hz, and 4000 Hz).

Furthermore, the DHI score was improved from an initial 52 score to 0 (no handicap at all) and the THI score improved 86 to 22 (mild handicap).

## Discussion

To the best of our knowledge, there are no published studies that evaluate the effectiveness of WCT in the management of inner ear pathologies or its effect on hearing in SSNHL. Hence, we are unable to compare our findings with other studies. Our presented case (48 years old, sudden moderate SSNHL, and no hearing improvement within two weeks of steroid use) suggested a poor prognosis. SSNHL patients with no improvement in hearing within two weeks are unlikely to recover; those with hearing loss for two to three months are likely to become permanently deaf [15]. Oral corticosteroids are the most common treatment for SSNHL with little supporting evidence for their effectiveness [15]. The integration of WCT in the treatment programs of SSNHL is promising. In our case, WCT improved the feeling of fullness after the first session and improved hearing loss, tinnitus, and dizziness after the completion of the WCT treatment regime (12 weeks). The observed effects of our case are unlikely to be due to steroid treatment because of the deterioration of the patient's hearing after treatment or due to the natural course of SSNHL and natural recovery since spontaneous recovery is usually within weeks of SSNHL [16]. In this case, we cannot give clear explanations as to how a complementary treatment, such as WCT, improved the patient’s symptoms. Whilst circulatory disturbances are the leading cause of the most sudden hearing loss conditions, cupping therapy can improve local blood circulation, remove oxidants, decrease oxidative stress, elevate blood oxygen, and provides a positive effect on hemodynamics [3, 17-19]. However, a placebo effect cannot be ruled out.

No local or systemic adverse events were reported in our case report. This is in agreement with previous research where adverse effects or adverse events related to cupping were infrequent, and most adverse effects were avoidable if trained personnel provided cupping therapy [20].

## Conclusions

We represent a case of SSNHL treated with WCT and conventional medical treatment. The substantial efficacy of WCT was reported in this case. Adding complementary medicine approaches, such as WCT, to the management protocol of SSNHL is worth trying if routine medical treatments alone fail. Further investigation with large well-designed quality trials to evaluate the efficacy and safety of WCT as a complementary treatment of SSHNL is highly recommended.
